# Elevated serum albumin-to-creatinine ratio as a protective factor on outcomes after heart transplantation

**DOI:** 10.3389/fcvm.2023.1210278

**Published:** 2023-09-07

**Authors:** Qiang Shen, Dingyi Yao, Yang Zhao, Xingyu Qian, Yidan Zheng, Li Xu, Chen Jiang, Qiang Zheng, Si Chen, Jiawei Shi, Nianguo Dong

**Affiliations:** ^1^ Department of Cardiovascular Surgery, Union Hospital, Tongji Medical College, Huazhong University of Science and Technology, Wuhan, China; ^2^ Key Laboratory of Organ Transplantation, Ministry of Education, Wuhan, China; ^3^ NHC Key Laboratory of Organ Transplantation, Wuhan, China; ^4^ Key Laboratory of Organ Transplantation, Chinese Academy of Medical Sciences, Wuhan, China

**Keywords:** heart failure, heart transplantation, albumin/creatinine ratio, prognosis, nomogram

## Abstract

**Background:**

The purpose of this study was to investigate the prognostic significance of serum albumin to creatinine ratio (ACR) in patients receiving heart transplantation of end-stage heart failure.

**Methods:**

From January 2015 to December 2020, a total of 460 patients who underwent heart transplantation were included in this retrospective analysis. According to the maximum Youden index, the optimal cut-off value was identified. Kaplan-Meier methods were used to describe survival rates, and multivariable analyses were conducted with Cox proportional hazard models. Meanwhile, logistic regression analysis was applied to evaluate predictors for postoperative complications. The accuracy of risk prediction was evaluated by using the concordance index (C-index) and calibration plots.

**Results:**

The optimal cut-off value was 37.54 for ACR. Univariable analysis indicated that recipient age, IABP, RAAS, BB, Hb, urea nitrogen, D-dimer, troponin, TG, and ACR were significant prognostic factors of overall survival (OS). Multivariate analysis showed that preoperative ACR (HR: 0.504, 95% = 0.352–0.722, *P* < 0.001) was still an independent prognostic factor of OS. The nomogram for predicting 1-year and 5-year OS in patients who underwent heart transplantation without ACR (C-index = 0.631) and with ACR (C-index = 0.671). Besides, preoperative ACR level was a significant independent predictor of postoperative respiratory complications, renal complications, liver injury, infection and in-hospital death. Moreover, the calibration plot showed good consistency between the predictions by the nomogram for OS and the actual outcomes.

**Conclusion:**

Our research showed that ACR is a favorable prognostic indicator in patients of heart transplantation.

## 1. Introduction

Heart failure is one of the most common cardiovascular manifestations (1). More than 26 million people worldwide are affected by heart failure (2). Heart transplantation (HTx) is the most effective and reliable treatment for end-stage heart failure (3). Over the past decades, the selection of HTx candidates and improvements in preoperative care have led to a steady improvement in outcomes after early HTx. Meanwhile, studies have shown that preoperative specific risk factors can predict survival after HTx, such as preoperative obesity, single-ventricle congenital heart disease, history of multiple thoracotomies, and renal replacement therapy (4–6). Therefore, it is very meaningful to study predictors of adverse HTx outcomes, as they may allow for closer monitoring and early intervention of patients at risk.

Human serum albumin is a key plasma protein and has important physiological functions such as immune regulation, endothelial stability, antioxidant effects, and binding to a variety of drugs, toxins, and other molecules (7). There is increasing evidence that serum albumin levels are closely associated with cardiovascular diseases, such as myocardial fibrosis, adverse pulsing aortic hemodynamics, heart failure, and coronary heart disease (8, 9). Furthermore, the serum albumin level before HTx is a useful marker for estimating post-transplantation survival (10). In addition, previous studies have pointed out that serum creatinine levels can affect prognosis in cardiac surgery (11). Importantly, glomerular filtration rate (GFR) is also often measured by serum creatinine level and can affect the prognosis of heart transplantation (9).

The definition of the ratio of serum albumin to creatinine (ACR) was proposed by Liu H (12). Recent studies have pointed out that it can be used to predict renal outcomes in diabetic patients. Meanwhile, ACR is also used in predicting the outcome of cardiovascular diseases, such as asymptomatic coronary artery disease and acute myocardial infarction (12–14). Nevertheless, its role in heart transplantation has not been investigated.

Therefore, in this retrospective study, we aimed to analyze the association of preoperative ACR levels with complications and overall survival (OS) after HTx.

## 2. Methods

### 2.1. Patients

**Figure 1:**
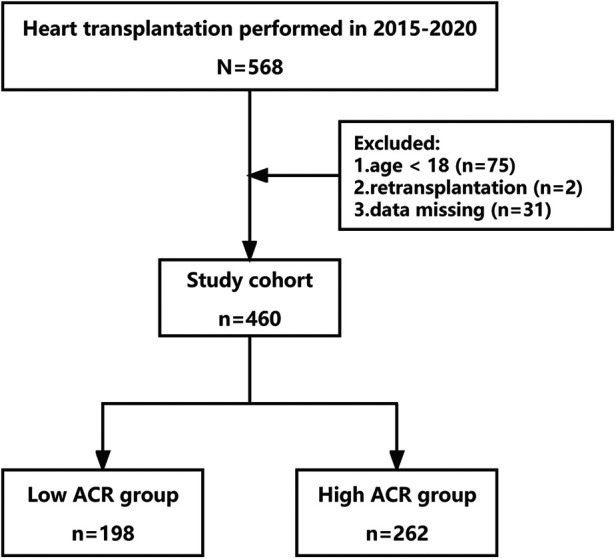
Inclusion and exclusion criteria.

Between 2015 and 2020, a total of 568 patients scheduled to undergo heart transplantation at Wuhan Union Medical College Hospital were included in this study. Age less than 18 years, retransplants, and patients with missing data were excluded. Ultimately 460 patients were recruited to the study and were divided into two groups of ACR > 37.54 (*n* = 262), and ACR ≤ 37.54 (*n* = 198), according to the optimal cut-off value of ACR (Figure 1). This retrospective study has been approved by the Committee of Tongji Medical College. The use and collection of patient data complied with the Declaration of Helsinki principles in our study.

### 2.2. Follow-up

Information on all survivors was collected through visits and telephone calls. OS was defined as the interval between surgery and death or last contact. The follow-up ended on December 31, 2020.

### 2.3. Demographic and clinical variables

Demographic variables of all patients included sex, age, blood type, body mass index (BMI), and diagnosis. Additionally, the recipients' information also included the history of smoking, diabetes mellitus, previous cardiac surgery, hypertension, left ventricular ejection fraction (LVEF), and waiting time. The preoperative therapy data included extracorporeal membrane oxygenation (ECMO), implantable intra-aortic balloon pump (IABP), renin-angiotensin-aldosterone system (RAAS) antagonist, beta-blockers (BB), calcium channel blocker (CCB) and diuretics. Preoperative blood biochemical indexes included hemoglobin (Hb), white blood cell count (WBC), blood platelet (PLT), albumin, creatinine (Cr), bilirubin, glutamic oxaloacetic transaminase (AST), alanine aminotransferase (ALT), low-density lipoprotein (LDL), troponin and triglyceride (TG). Preoperative hematological and biochemical indicators are the first results of the first admission of a heart transplant patient.

### 2.4. Postoperative clinical events

We compared several postoperative clinical events between the high and low ACR groups, including postoperative ICU stay time, total postoperative hospital stay time, the use of postoperative CRRT, IABP, and ECMO, respiratory complications, neurological complications, hematological complications, hyperglycemia, hypertension, infection, renal injury, liver injury, septic shock, secondary thoracotomy, and death in hospital.

### 2.5. Definition

As previously reported in the literature, ACR was calculated from the ratio of serum albumin (mg/dl) to creatinine (mg/dl) (15). Postoperative infection is defined as an infection of soft tissues and organs after surgery and arises when the balance between host defense mechanisms and bacterial load or virulence is disrupted (16, 17).

### 2.6. Statistical analysis

In this study, continuous variables are expressed as mean ± standard deviation or median [interquartile range] according to their normality, whereas categorical variables are expressed as percentages. Different ACR groups were compared at baseline concerning participants' characteristics and outcome measures using Mann–Whitney U tests for continuous variables, which were tested to be non-normal distributions, and *χ*^2^ tests for categorical variables. The Cox proportional hazards regression model was used to determine independent predictors of mortality after heart transplantation. Besides, univariate and multivariable logistic regression was employed to identify predictors of postoperative clinical events. Survival analysis was performed using the Kaplan-Meier method, and significance was assessed by the log-rank test. Two-tailed *P* values < 0.05 were considered significant. The statistical analysis was performed with SPSS 23.0 and R-software v.4.2.1.

## 3. Results

### 3.1. Population characteristics

**Figure 2:**
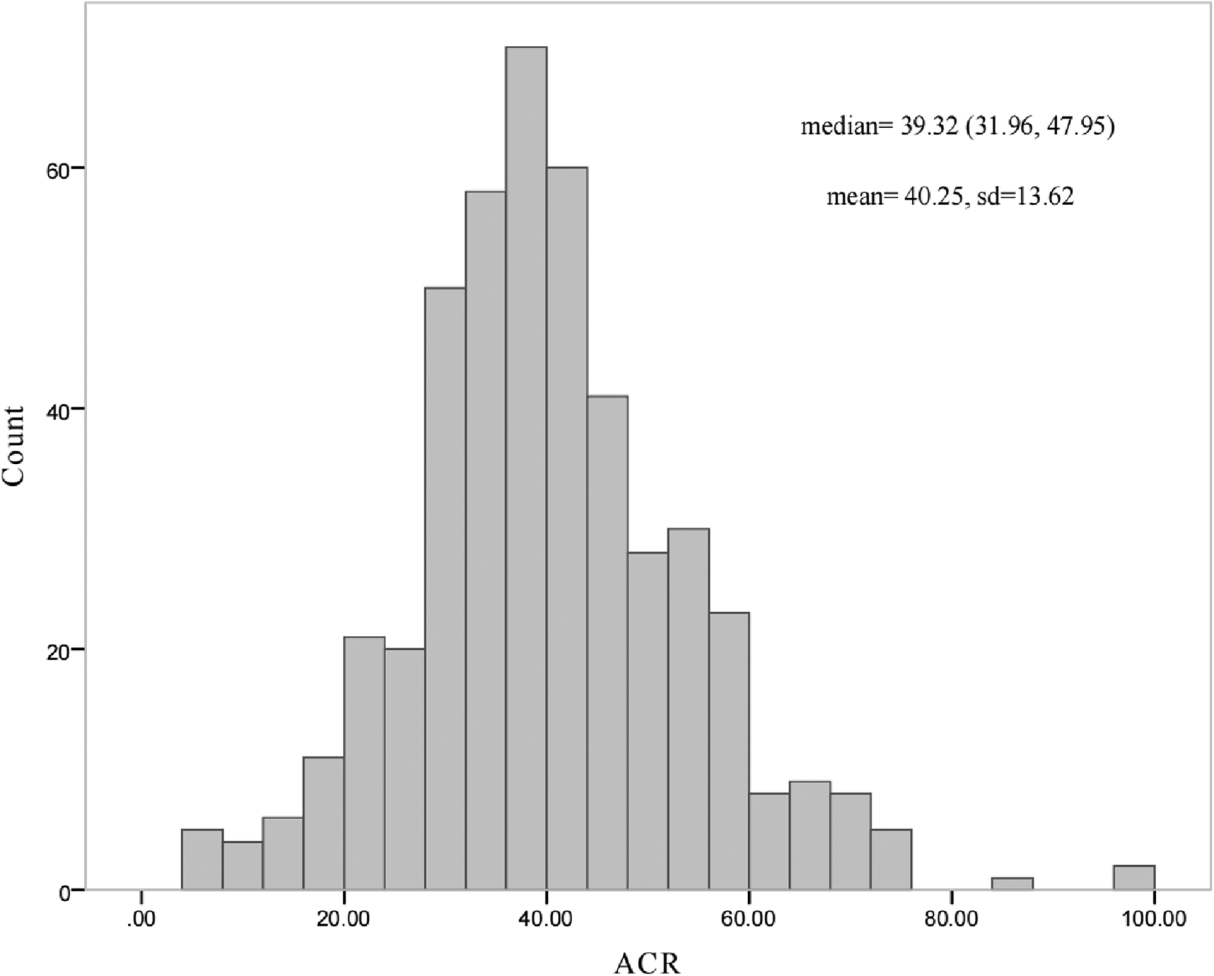
Histogram plot of ACR. ACR, serum albumin to creatinine ratio.

According to the inclusion and exclusion criteria, a total of 460 patients were included in the study. The median age was 50.00 years (38.00–57.00) and 359 (78%) of the patients were male. The median ACR of the samples was 39.32 (31.96–47.95). The histogram curve of ACR distribution is shown in Figure 2.

### 3.2. The optimal cut-off values for estimating prognosis

**Figure 3:**
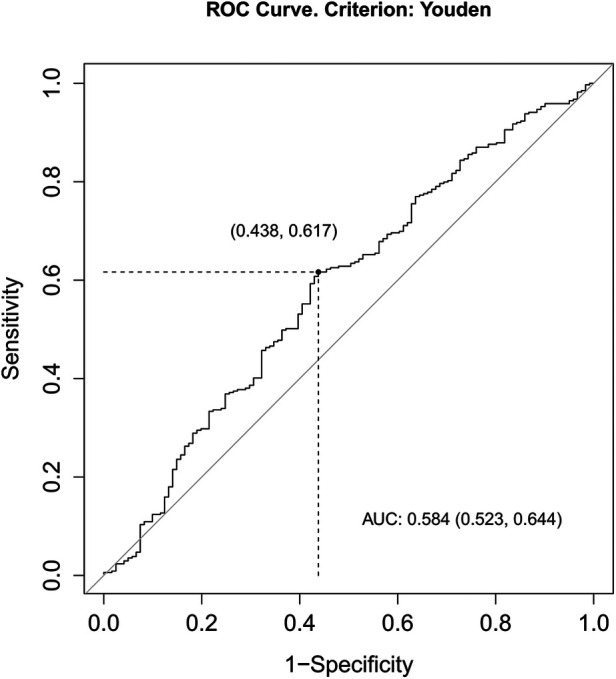
Receiver operating characteristic curves for pretreatment ACR.

The association between ACR and survival is shown in the ROC curve (Figure 3). During the process, the area under the curve (AUC) for survival was 0.584 (*P* = 0.006). According to the maximum Youden index, it revealed that 37.54 was an optimal cut-off value of the ACR index for predicting the survival rate. By the optimal cut-off value of ACR, the patients were divided into two groups (high, ≥37.54, and low, <37.54).

### 3.3. Characteristics of patients

Table 1 showed baseline patients' characteristics based on ACR. Patients with higher ACR levels tended to have a less proportion of males (*P* < 0.001) and chronic kidney disease (*P* < 0.001), younger age (*P* < 0.001), less hypertension (*P* = 0.017), more use of spironolactone (*P* = 0.002) and thiazides (*P* = 0.010), higher preoperative level of blood platelet (*P* = 0.033) and albumin (*P* < 0.001), lower preoperative levels of white blood cells (*P* = 0.001), creatinine (*P* < 0.001) and AST (*P* = 0.025), and a different proportions of Donor/recipient sex (*P* = 0.001).

**Table 1:** Baseline patient characteristics based on ACR.

Recipient		Low (<37.54)	High (>37.54)	*P*
	Male, %	173 (87.4)	186 (71.0)	<0.001
	Age, years	50.50 [39.25, 57.00]	46.00 [34.00, 53.00]	<0.001
	BMI, Kg/m^2^	23.39 [20.50, 25.51]	22.73 [19.63, 25.39]	0.079
	History of smoking, %	87 (43.9)	105 (40.1)	0.405
	Diagnosis, %			0.091
	Non-Ischemic cardiomyopathy	134 (67.7)	156 (59.5)	
	Ischemic cardiomyopathy	38 (19.2)	48 (18.3)	
	Congenital	5 (2.5)	14 (5.3)	
	Others	21 (10.6)	44 (16.8)	
	Diabetes mellitus, %	27 (18.2)	39 (18.6)	0.937
	Previous cardiac surgery, %	53 (26.8)	76 (29.0)	0.596
	Hypertension, %	39 (21.8)	30 (12.9)	0.017
	chronic kidney disease, %	25 (12.7)	3 (1.2)	<0.001
	interventricular septum, cm	0.90 [0.80, 1.00]	0.90 [0.80, 1.00]	0.568
	LVEF, %	16.10 [12.00, 26.90]	16.25 [12.00, 22.38]	0.412
	Waiting time, days	26.00 [19.00, 37.00]	27.00 [19.00, 35.00]	0.700
	Recipient blood-type, %			0.621
	A	62 (31.3)	94 (35.9)	
	B	56 (28.3)	73 (27.9)	
	O	69 (34.8)	78 (29.8)	
	AB	11 (5.6)	17 (6.5)	
	Preoperative Therapy			
	ECMO, %	4 (2.0)	2 (0.8)	0.410
	IABP, %	4 (2.0)	4 (1.5)	0.730
	RAAS antagonist, %	84 (42.4)	102 (38.9)	0.450
	BB, %	135 (68.2)	179 (68.3)	0.975
	CCB, %	17 (15.7)	16 (10.9)	0.254
	Loop diuretics, %	188 (96.9)	247 (96.1)	0.650
	Spironolactone, %	142 (74.0)	216 (85.7)	0.002
	Thiazides, %	4 (2.2)	19 (8.0)	0.010
	Preoperative Blood Index			
	Hb, g/L	132.50 [120.25, 150.50]	140.00 [129.00, 148.00]	0.115
	WBC, G/L	6.70 [5.03, 8.76]	5.89 [4.56, 7.52]	0.001
	PLT, G/L	163.00 [129.75, 212.50]	178.50 [139.00, 222.25]	0.033
	Albumin, mg/dl	37.90 [35.83, 40.58]	40.70 [37.73, 42.90]	<0.001
	Creatinine, mg/dl	110.40 [97.80, 129.20]	76.45 [67.93, 85.98]	<0.001
	Bilirubin, mg/dl	21.50 [14.75, 33.15]	21.15 [13.83, 33.88]	0.267
	AST, IU/L	29.50 [21.00, 43.50]	28.00 [20.00, 35.50]	0.025
	ALT, IU/L	30.50 [17.00, 57.00]	25.00 [16.25, 42.00]	0.065
	LDL, mmol/L	2.19 [1.71, 2.85]	2.26 [1.72, 2.81]	0.982
	Troponin, ng/L	29.20 [0.01, 101.90]	16.00 [0.00, 95.23]	0.310
	TG, mmol/L	0.99 [0.73, 1.24]	1.09 [0.82, 1.49]	0.362
Donor	Age, years	38.00 [26.00, 45.00]	36.00 [24.00, 45.50]	0.599
	Male, %	175 (88.4)	225 (85.9)	0.429
	Ischemia time, min	358.00 [297.75, 396.25]	345.00 [290.50, 392.50]	0.257
Donor/recipient	Blood-type same, %	125 (63.1)	163 (62.2)	0.840
	Donor/recipient BMI	0.97 [0.86, 1.12]	1.00 [0.88, 1.17]	0.135
	Donor/recipient age	0.74 [0.53, 0.89]	0.77 [0.55, 1.00]	0.125
	Donor/recipient sex, %			0.001
	Male/male	157 (79.3)	169 (65.0)	
	Male/female	18 (9.1)	54 (20.8)	
	Female/male	16 (8.1)	16 (6.2)	
	Female/female	7 (3.5)	21 (8.1)	

Continuous variables are presented as median and interquartile range. Categorical variables are presented as number and percentage. *P* values are 2-sided, with *P* < 0.05 considered statistically significant.

BMI, body mass index; LVEF, left ventricular ejection fraction; ECMO, extracorporeal membrane oxygenation; IABP, implantable intra-aortic balloon pump; RAAS, renin-angiotensin-aldosterone system; BB, beta-blockers; CCB, calcium channel blocker; Hb, hemoglobin; WBC, white blood cell count; PLT, blood platelet; Cr, creatinine; AST, glutamic oxaloacetic transaminase; ALT, alanine aminotransferase; LDL, low-density lipoprotein; TG, triglyceride.

### 3.4. Univariate and multivariate analyses of Os

**Figure 4:**
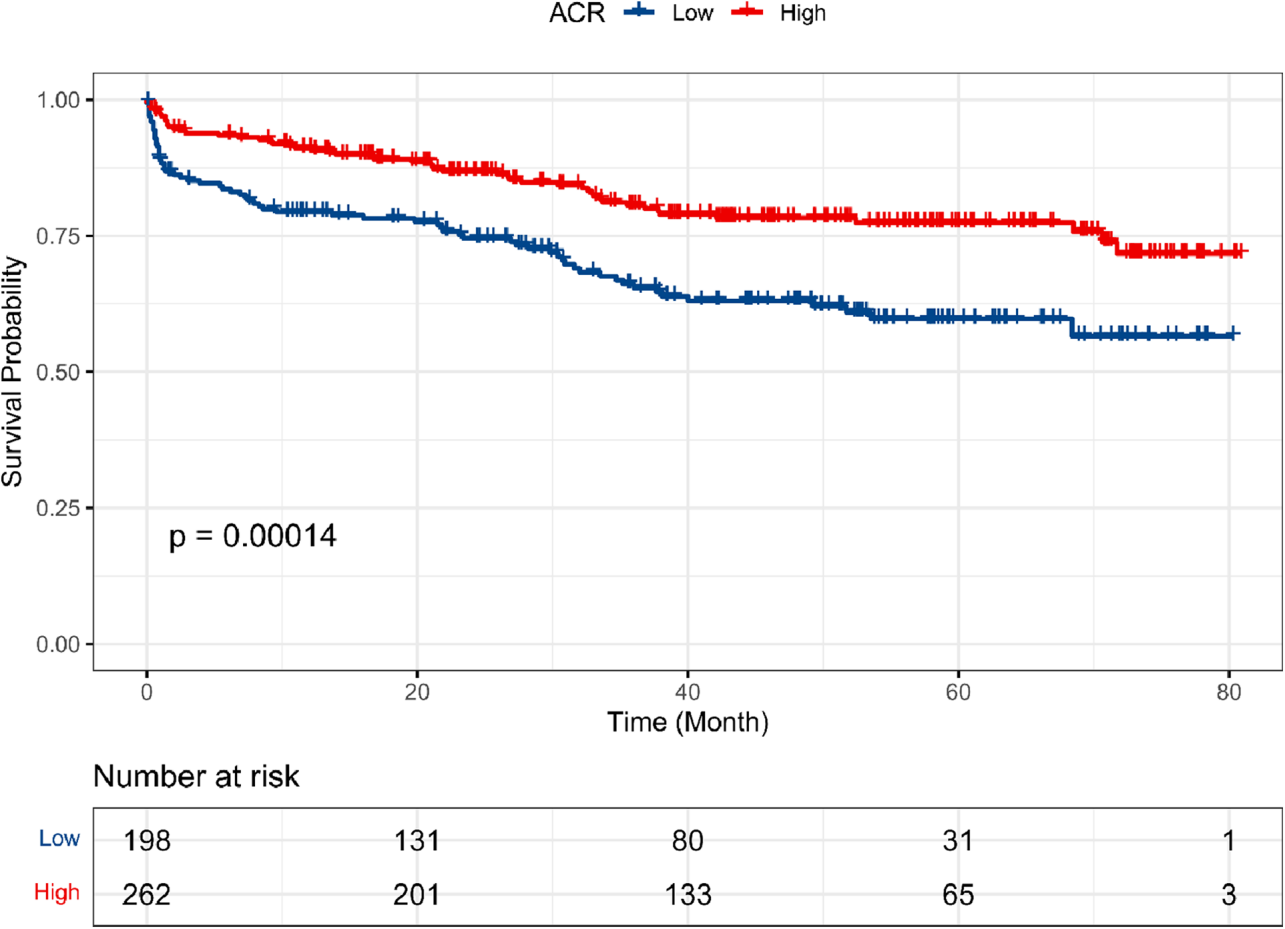
Kaplan–Meier survival curves for OS according to ACR. The low-ACR value group. Exhibited worse OS than high-ACR value group (*P* < 0.001).

At the end of follow-up, 121 (26.3%) patients had died and 339 (73.7%) patients were alive. The Kaplan-Meier curve (Figure 4) shows the association between OS and ACR for all patients. Patients with high ACR levels had better survival than those with low ACR levels (*P* < 0.001). To identify the risk factors affecting postoperative OS, the Cox proportional hazard model was applied to the analysis. Univariable analysis indicated that recipient age (*P* < 0.001), the preoperative use of IABP (*P* = 0.004), RAAS (*P* = 0.001), and BB (*P* = 0.016), preoperative level of Hb (*P* < 0.001), BUN (*P* = 0.004), D-dimer (*P* = 0.018), Troponin (*P* = 0.014), TG (*P* = 0.043) and ACR (*P* < 0.001) were significant prognostic factors of OS (Table 2). Nextly, significant prognostic factors identified by univariate analysis were entered into the multivariate Cox proportional hazards model. The results showed that recipient age (*P* = 0.005), the use of IABP (*P* = 0.008) and RAAS (*P* = 0.014), preoperative level of D-dimer (*P* = 0.041) and ACR (*P* = 0.019) were significant independent predictors of OS (Table 2).

**Table 2:** Univariate and multivariate Cox proportional hazards regression models for overall survival in patients with heart transplantation.

Variables	Univariate analysis		Multivariate analysis	
	HR (95%CI)	*P* value	HR (95%CI)	*P* value
Sex	1.458 (0.981–2.168)	0.062		
Age, years	1.030 (1.014–1.047)	<0.001	1.023 (1.006–1.040)	0.005
Recipient BMI, Kg/m^2^	1.017 (0.989–1.046)	0.228		
History of smoking	0.778 (0.537–1.128)	0.185		
History of heavy drinking	0.740 (0.475–1.152)	0.182		
Waiting time, days	0.993 (0.980–1.005)	0.258		
Diagnosis		0.458		
Non-Ischemic cardiomyopathy	Reference			
Ischemic cardiomyopathy	0.755 (0.447–1.273)	0.291		
Congenital	0.558 (0.176–1.768)	0.321		
others	1.143 (0.700–1.865)	0.594		
Recipient blood-type		0.061		
A	Reference			
B	1.293 (0.804–2.080)	0.290		
O	1.680 (1.085–2.602)	0.020		
AB	0.664 (0.236–1.872)	0.439		
Donor/recipient BMI	1.528 (0.779–2.997)	0.218		
Donor/recipient age	0.684 (0.418–1.121)	0.132		
Donor/recipient sex		0.149		
Male/male	Reference			
Male/female	1.621 (1.031–2.548)	0.036		
Female/male	1.481 (0.765–2.865)	0.244		
Female/female	1.385 (0.694–2.765)	0.356		
Donor/recipient blood-type same	0.844 (0.581–1.227)	0.374		
Diabetes mellitus	1.366 (0.814–2.292)	0.237		
Previous cardiac surgery	1.314 (0.899–1.919)	0.158		
hypertension	0.981 (0.584–1.647)	0.942		
Ischemia time, min	1.000 (0.999–1.002)	0.590		
Preoperative Therapy				
ECMO	1.283 (0.178–9.227)	0.805		
IABP	4.333 (1.586–11.839)	0.004	4.086 (1.440–11.600)	0.008
ICD	1.182 (0.551–2.536)	0.668		
CRTD	0.856 (0.272–2.696)	0.791		
RAAS	0.527 (0.357–0.780)	0.001	0.593 (0.391–0.899)	0.014
BB	0.641 (0.446–0.922)	0.016	0.837 (0.568–1.234)	0.370
CCB	1.174 (0.581–2.374)	0.655		
Loop diuretics	0.725 (0.296–1.778)	0.483		
Spironolactone	0.826 (0.526–1.298)	0.407		
Thiazides	1.482 (0.687–3.201)	0.316		
Preoperative Blood Index				
Hb, g/L	0.989 (0.982–0.995)	<0.001	0.993 (0.985–1.000)	0.064
WBC, G/L	1.012 (0.988–1.037)	0.323		
Bilirubin, mg/dl	1.005 (0.997–1.013)	0.203		
BUN, mmol/L	1.054 (1.017–1.091)	0.004	1.004 (0.958–1.053)	0.856
AST, IU/L	1.000 (1.000–1.001)	0.216		
ALT, IU/L	1.000 (1.000–1.001)	0.903		
LDL, mmol/L	1.158 (0.958–1.399)	0.130		
D-dimer, mg/L	1.028 (1.005–1.052)	0.018	1.025 (1.001–1.049)	0.041
Troponin, ng/ml	1.000 (1.000–1.000)	0.014	1.000 (1.000–1.000)	0.094
TG, mmol/L	0.737 (0.549–0.991)	0.043	0.825 (0.606–1.122)	0.220
ACR level	0.504 (0.352–0.722)	<0.001	0.617 (0.412–0.923)	0.019

95%CI, 95% confidence interval; HR, hazard ratio.

Continuous variables are presented as median and interquartile range. Categorical variables are presented as numbers and percentages. *P* values are 2-sided, with *P* < 0.05 considered statistically significant.

BMI, body mass index; ECMO, extracorporeal membrane oxygenation; IABP, implantable intra-aortic balloon pump; ICD, implantable cardioverter defibrillator; CRTD, cardiac resynchronization therapy defibrillator; RAAS, renin-angiotensin-aldosterone system; BB, beta-blockers; CCB, calcium channel blocker; Hb, hemoglobin; WBC, white blood cell count; PLT, blood platelet; Cr, creatinine; AST, glutamic oxaloacetic transaminase; ALT, Alanine aminotransferase; LDL, low-density lipoprotein; TG, triglyceride; ACR, the ratio of serum albumin to creatinine.

### 3.5. Univariate and multivariate analysis of postoperative clinical events

A total of 17 surgery-related adverse clinical events occurred during the in-hospital posttransplant period, listed in Table 3. The result showed that lower levels of ACR tended to lead to more use of postoperative CRRT (*P* < 0.001) and IABP (*P* = 0.001), more respiratory complications (*P* = 0.001), liver injury (*P* = 0.005), kidney injury (*P* = 0.001), postoperative infection (*P* = 0.001), septic shock (*P* = 0.012), and in-hospital death (*P* = 0.001). We next performed univariate logistic regression analysis for these adverse clinical events (Supplementary Schedules S1–S8), and then the factors with *P* < 0.05 in univariate analysis were applied to multivariate logistic regression analysis. The results showed that lower preoperative ACR level was a significantly independent predictor of respiratory complications (*P* = 0.043), renal complications (*P* = 0.007), liver injury (*P* = 0.019), postoperative infection (*P*= 0.003) and in-hospital death (*P* = 0.028) (Table 4). What's more, higher age, less preoperative RAAS use and lower TG were independent risk factors of respiratory complication. Women and less RAAS use were independent risk factors of kidney injury. And less preoperative RAAS use was also associated with postoperative liver injury. Longer waiting time, diabetes mellitus and preoperative IABP were independently associated with in-hospital death. Moveover, smoking was an independent risk factor postoperative infection.

**Table 3:** Early postoperative events in the in-hospital post-transplant period by pretransplant ACR.

Variables	All	Low (<37.54)	High (>37.54)	*P*
Postoperative ICU stay, hours	216 [162,294]	223 [168, 331]	213 [159, 275]	0.326
Total postoperative stay, days	35 [26,49]	35 [27, 54]	35 [26, 47]	0.076
Postoperative CRRT (%)	67 (14.6)	43 (22.3)	24 (9.3)	<0.001
Postoperative IABP (%)	191 (41.5)	99 (50.8)	92 (35.5)	0.001
Postoperative ECMO (%)	33 (7.2)	19 (9.9)	14 (5.4)	0.072
Respiratory complication (%)	278 (60.4)	137 (69.2)	141 (53.8)	0.001
Neurological complications (%)	33 (7.2)	17 (8.6)	16 (6.1)	0.308
Hematological complications (%)	19 (4.1)	11 (5.6)	8 (3.1)	0.182
Kidney injury (%)	73 (15.9)	44 (22.2)	29 (11.1)	0.001
Liver injury (%)	35 (7.6)	23 (11.6)	12 (4.6)	0.005
Infection (%)	234 (50.9)	116 (64.4)	118 (48.6)	0.001
Hyperglucosemia (%)	42 (9.1)	20 (10.1)	22 (8.4)	0.530
Hypertension (%)	25 (5.4)	11 (5.6)	14 (5.3)	0.921
Septic shock (%)	13 (2.8)	10 (5.1)	3 (1.1)	0.012
Secondary thoracotomy (%)	19 (4.1)	11 (5.7)	8 (3.1)	0.178
Acute rejection (%)	9 (2.0)	3 (1.5)	6 (2.3)	0.805
Death (%)	24 (5.2)	18 (9.1)	6 (2.3)	0.001

Continuous variables are presented as median and interquartile range. Categorical variables are presented as numbers and percentages. *P* values are 2-sided, with *P* < 0.05 considered statistically significant.

CRRT, continuous renal replacement therapy; IABP, implantable intra-aortic balloon pump; ECMO, extracorporeal membrane oxygenation; sACR, the ratio of serum albumin to creatinine.

**Table 4:** Multivariable analysis for early postoperative events following heart transplantation.

Early Postoperative Events	ACR	
	OR (95%CI)	*P* value
Prolonged Postoperative ICU Stay	0.835 (0.526–1.327)	0.446
Respiratory Complications	0.629 (0.402–0.985)	0.043
Kidney Complications	0.424 (0.227–0.790)	0.007
Infection	0.540 (0.358–0.816)	0.003
Liver Injury	0.394 (0.182–0.856)	0.019
Septic Shock	0.257 (0.059–1.117)	0.070
In-hospital death	0.250 (0.073–0.863)	0.028

ACR, the ratio of serum albumin to creatinine.

### 3.6. Prognostic nomogram for OS

**Figure 5:**
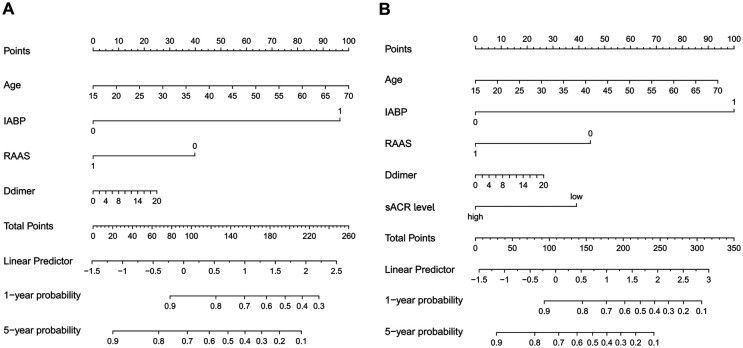
The nomogram for predicting 1-year and 5-year OS in patients who underwent HTx without ACR [(**A**), C-index = 0.631] and with ACR [(**B**), C-index = 0.671].

**Figure 6:**
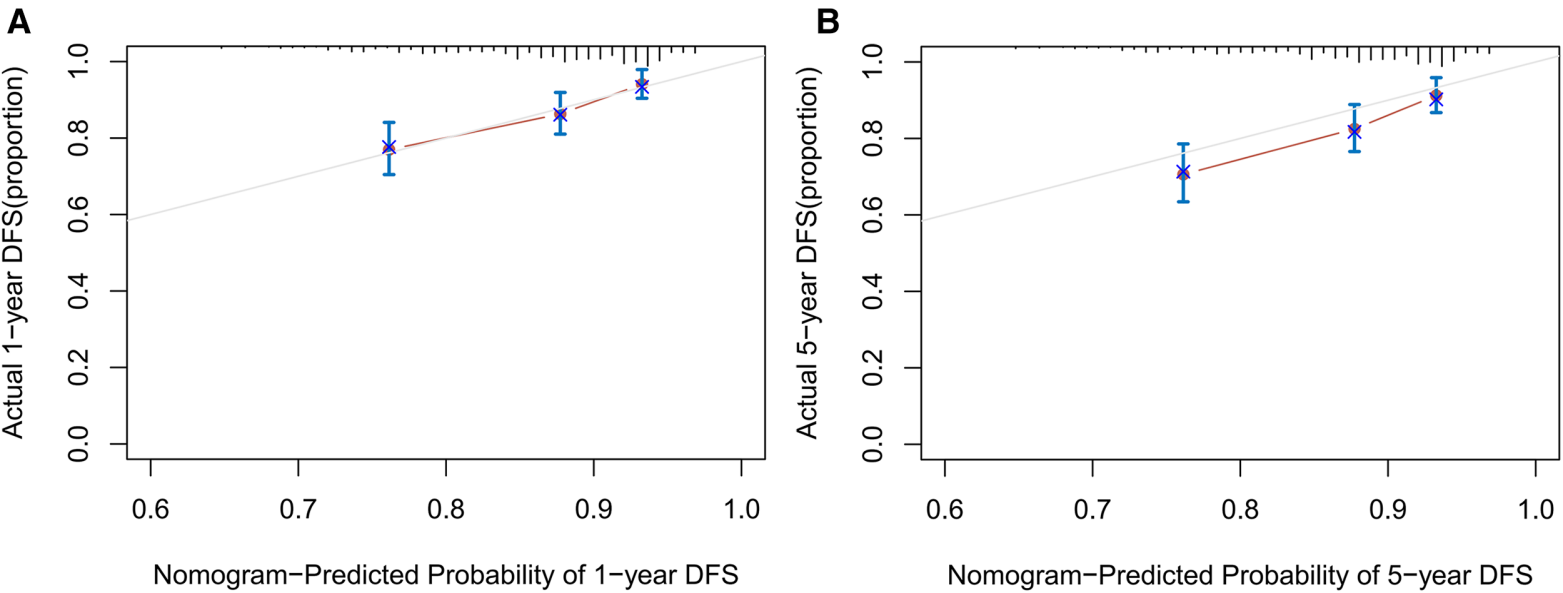
The calibration curves for predicting 1-year (**A**) and 5-year (**B**) OS of patients after HTx. Each point in the plot represents a group of patients, with the nomogram predicted probability of survival shown on x-axis and actual survival proportion shown on y-axis.

A nomogram (Figure 5) integrating was constructed based on five prognostic variables (age, IABP, RAAS, D-dimer, and ACR) from the univariate and multivariate Cox regression results. A Nomogram with ACR had a concordance index (C-index) of 0.671 compared with 0.631 without ACR. The calibration plot (Figure 6) showed good consistency between the nomogram predictions and actual observations of survival at 1- or 5- years.

## 4. Discussion

Heart failure is a common disease and the best treatment for patients with end-stage heart failure is HTx (1, 3). Meanwhile, many factors can predict the outcomes of HTx. For example, recent studies have pointed out that ACR as a preoperative indicator can predict the prognosis of cardiovascular disease (12–14). In this retrospective study, we evaluated the effect of ACR on the prognosis of HTx and found that high levels of ACR were a protective factor after HTx. Meanwhile, five possible prognostic factors (age, the use of IABP, RAAS, D-dimer, and ACR) were identified according to multivariable Cox regression analysis. Furthermore, multivariate logistic regression analysis showed that low levels of ACR are associated with several post-transplant complications, including respiratory complications, renal complications, liver injury, and in-hospital death. In addition, a visual nomogram was created in light of clinical variables and ACR, which helped to improve individual prognosis prediction accuracy.

Albumin is an important serum protein and has a wide range of physiological functions, such as immune regulation, endothelial stabilization, antioxidant effects, and binding to a variety of drugs, toxins, and other molecules (7). Albumin can also be used as a biomarker for many diseases, such as cancer, ischemia, obesity, severe acute graft-vs.-host disease, and diseases requiring monitoring of glycemic control (18). Specifically, numerous studies have demonstrated a strong association between serum albumin levels and the prognosis of cardiovascular diseases, such as atherosclerosis, myocardial infarction, and heart failure (19, 20). Moreover, Tomoko et al. proposed the effect of pre-transplant albumin levels on 1-year survival after heart transplantation in a retrospective study (21).

Creatinine was used as an index of renal function, which reflects not only renal excretion but also creatinine production (22, 23). In most genome-wide association studies, creatinine-based assessment of renal function (eGFR crea) has been used to define renal disease (24). Since the link between chronic kidney disease and cardiovascular disease was first described, numerous research suggested that chronic kidney disease greatly increases the risk of cardiovascular disease. This is partly because abnormal renal function leads to abnormal blood pressure, lipids, inflammatory responses, and increased activity of the renin-angiotensin system (25). Apart from preoperative renal disease, acute kidney injury is often a common complication after heart transplantation (26). It is associated with increased short- and long-term morbidity and mortality (27). Therefore, an important next step will be to investigate preoperative factors affecting AKI after heart transplantation.

The possibility of the correlation between ACR and the prognosis of HTx might be as follows: Firstly, Low serum albumin is often a marker of poor liver function (28). Likewise, in our study, we found that the preoperative ACR level was closely related to the occurrence of postoperative liver injury. In addition, albumin can improve the prognosis of heart transplantation by regulating systemic inflammatory response and immune response (7). Physiological concentrations of albumin attenuate inflammation by selectively inhibiting TNF*α*-induced upregulation of VCAM-1 expression and monocyte adhesion (29). Moreover, albumin inhibits histone-induced platelet aggregation and thrombus formation by binding to histones (30). These help explain why a low preoperative ACR level is associated with a higher incidence of septic shock. Besides, it is well established that oxidative stress is a common risk factor in various diseases, such as diabetes, inflammation, and cardiovascular disease. Oxidative stress generated by excessive reactive oxygen species (ROS) promotes cardiovascular disease (31). For example, starting point of atherosclerosis is considered to be oxidative stress, which facilitates key molecular events, such as oxidative modification of lipoproteins and phospholipids, endothelial cell activation, and macrophage infiltration/activation (32). Importantly, albumin exerts its antioxidant function by binding and neutralizing free metals such as copper and iron at its N-terminal site and specifically regulates cellular glutathione levels (7). Secondly, creatinine is a waste product of muscle metabolism (33). Produced at a continuous rate by creatine metabolism and excreted without tubular reabsorption, it is used as a marker of GFR (34). It is well-known that the reduction of GFR is now a recognized risk factor for cardiovascular disease (CVD) and chronic kidney disease (35, 36). Similarly, our results showed that low preoperative ACR levels are associated with a higher risk for postoperative Kidney Complications and In-hospital death. Besides, many studies have pointed out that chronic kidney disease often leads to dyslipidemia and inflammation, which leads to the hardening of the aorta and the reduction of coronary reserve (25, 37). Furthermore, kidney disease may cause remodeling of the ventricle through hypertension, renal anemia, and vascular stiffness, thus leading to hypertrophy of the left ventricle (25, 37). These biological processes may help explain associations between the level of ACR and the prognosis of HTx.

As far as we know, it is the first study to assess the role of ACR in HTx outcomes. Our study confirmed that preoperative ACR was a novel and promising indicator that independently predicts the outcomes of HTx. This investigation gives us some clues about preoperative interventions to reduce postoperative complications, such as albumin supplements (38–40).

However, there are some limitations of this study that should be considered. Firstly, this is a retrospective and observational study, which ignores the progression of the disease and has inherent risks of information bias. Secondly, the sample size of this study was small (*n* = 460) and the follow-up period was relatively short. Thirdly, preoperative frailty may have effect on the study results. Future studies about heart transplantation should consider this issue. What's more, null of the subjects in our study developed early graft failure, so the evaluation of early graft failure could not be performed. Lastly, we did not obtain cytokines, markers of glucose metabolism, or serum inflammatory markers, all of which may affect the prognosis of heart transplantation.

## Data Availability

The data analyzed in this study is subject to the following licenses/restrictions: a portion of the data, models, or code generated or used during the study is proprietary or confidential in nature and may only be provided with restrictions (e.g., anonymized data). Requests to access these datasets should be directed to Dong N, 1986xh0694@hust.edu.cn.
